# Seasonality in trauma admissions – Are daylight and weather variables better predictors than general cyclic effects?

**DOI:** 10.1371/journal.pone.0192568

**Published:** 2018-02-09

**Authors:** Jo Røislien, Signe Søvik, Torsten Eken

**Affiliations:** 1 Faculty of Health Sciences, University of Stavanger, Stavanger, Norway; 2 Department of Research, Norwegian Air Ambulance Foundation, Drøbak, Norway; 3 Department of Anaesthesiology, Akershus University Hospital, Lørenskog, Norway; 4 Institute of Clinical Medicine, Faculty of Medicine, University of Oslo, Oslo, Norway; 5 Department of Anaesthesiology, Oslo University Hospital, Oslo, Norway; University of Oxford, UNITED KINGDOM

## Abstract

**Background:**

Trauma is a leading global cause of death, and predicting the burden of trauma admissions is vital for good planning of trauma care. Seasonality in trauma admissions has been found in several studies. Seasonal fluctuations in daylight hours, temperature and weather affect social and cultural practices but also individual neuroendocrine rhythms that may ultimately modify behaviour and potentially predispose to trauma. The aim of the present study was to explore to what extent the observed seasonality in daily trauma admissions could be explained by changes in daylight and weather variables throughout the year.

**Methods:**

Retrospective registry study on trauma admissions in the 10-year period 2001–2010 at Oslo University Hospital, Ullevål, Norway, where the amount of daylight varies from less than 6 hours to almost 19 hours per day throughout the year. Daily number of admissions was analysed by fitting non-linear Poisson time series regression models, simultaneously adjusting for several layers of temporal patterns, including a non-linear long-term trend and both seasonal and weekly cyclic effects. Five daylight and weather variables were explored, including hours of daylight and amount of precipitation. Models were compared using Akaike’s Information Criterion (AIC).

**Results:**

A regression model including daylight and weather variables significantly outperformed a traditional seasonality model in terms of AIC. A cyclic week effect was significant in all models.

**Conclusion:**

Daylight and weather variables are better predictors of seasonality in daily trauma admissions than mere information on day-of-year.

## Introduction

Trauma accounts for more deaths and disabilities worldwide than malaria, tuberculosis and HIV/AIDS combined [1]. Predicting the burden of trauma admissions over time could contribute to better planning of trauma care and thus be of considerable benefit. Predictable variations in the number of trauma admissions occur throughout the week, with an increase on weekends due to leisure activities [2]. Seasonal effects with a higher number of trauma admissions in spring and summer have also regularly been observed [3, 4].

While seasonality is an observable and significant predictor of trauma admissions it is not an effect in its own right, but rather the collective term we attach to the cyclic changes of various daylight and weather variables throughout the year. Seasonal fluctuations in daylight hours, temperature and weather affect social and cultural practices such as choice of transportation mode (e.g., car versus bike or motorcycle), sports and leisure, and overall activity level [5]. Moreover, individual neuroendocrine rhythms are affected by the amount of daylight (photoperiodism). This may ultimately modify behaviour and potentially predispose to trauma.

In healthy Danish males, a three-week bright-light intervention during winter season enhanced the functional MRI neural response to risk-taking in a card gambling game [6]. A nationwide US material of Emergency Department admissions for suicide attempts and self-harm showed a pronounced peak from March to May [7], similar to the pattern of non-voluntary psychiatric admissions in Italy [8]. For bipolar disorder a close association exists between increase in daylight hours and both the onset of disease and subsequent hospital admissions [9, 10]. In animals, absolute hours of daylight, but also the change in daylight hours from the previous day, affect behaviour via the retinal–hypothalamic–pineal axis [11]. Breeding-related and migratory behaviour is particularly affected. In a Koala wildlife facility, trauma admissions of young males predominated during spring and summer, typical mechanisms being car accidents and dog attacks during roaming and falls from trees during fights with other males [12]. A similar over-representation of young males is found in the Norwegian trauma population [13].

The association between weather and trauma has been studied previously [2, 4, 14–16]. To explore whether the observed seasonality in daily trauma admissions could be explained by daylight and weather variables, we applied an Additive Fourier Poisson time series regression model recently suggested for the analysis of seasonality in aggregated monthly suicide data [17]. The model simultaneously adjusts for both cyclic and non-cyclic short term and long term temporal phenomena, without the need for categorization. Covariates can easily be added to the model, and are used for standard modelling of the effect of various daylight and weather variables.

The aim of this study was to explore if various daylight and weather variables could explain the observed seasonality in trauma admissions, and to compare various statistical models.

## Materials and methods

### Data material

In this retrospective observational study we obtained anonymised data from the Oslo University Hospital (OUH) Trauma Registry on daily number of trauma admissions in the 10-year period from 01.01.2001 through 31.12.2010. Patients were allocated to a given date if they arrived between 06:00 local time that day and 05:59 the next day. OUH Ullevål is a major trauma referral hospital covering a geographical area with 2.8 million inhabitants in the South-Eastern part of Norway. Detailed information on the inclusion criteria for the OUH Trauma Registry can be found elsewhere [18]. Only aggregated data were used in the analyses. The study was approved and the need for written informed consent waived by the OUH Privacy Ombudsman for Research (29.09.2011, subject number 2011/16939), on behalf of the Norwegian Data Protection Authority and the Regional Committee for Medical Research Ethics.

The focus of the study was natural phenomena, leaving out local cultural phenomena such as religious holidays and independence day celebrations. The following five daylight and weather variables were included in the regression models: 1) Daylight hours, defined as the number of hours the Sun is above the horizon in Oslo, as calculated by The United States Naval Observatory Astronomical Applications Department [19]. 2) Difference in number of daylight hours from previous day, as a measure of the daylight gradient. 3) Hours of actual sunshine, 4) Mean temperature, and 5) mm precipitation, as collected from The Norwegian Meteorological Institute [20]. Amount of precipitation was both zero inflated and heavily skewed, and was therefore categorized into four categories in the analyses; 0, (0, 5], (5, 10] and (10, →) mm precipitation/day.

Precipitation data was measured in the morning at 07:00 local time, reflecting the amount of precipitation over the previous 24 hours. For the statistical analysis, precipitation measurements were therefore shifted one day backwards to match the actual day of the precipitation. A total of 1506 (41.2%) of precipitation observations were missing. However, comparing the precipitation data with those from two nearby weather stations revealed that missing tended to imply no precipitation. Missing values for precipitation was thus imputed with the value zero.

Hours of actual sunshine was missing for 173 (4.74%) of the observations. In addition, for 13 days (0.4%), hours of actual sunshine was reported as higher than the number of daylight hours. For these 13 days, hours of actual sunshine was substituted with missing, resulting in a total of 186 days (5.1%) with missing values for hours of actual sunshine. Mean temperature also had one missing value. This amount of missing data is generally low enough for complete case analysis to be stable, but since objective model comparison criteria need identically sized datasets in order to be comparable, we performed single imputation for hours of actual sunshine and mean temperature using multivariate imputation by chained equations (MICE) [21, 22].

### Statistical methods

To the time series of daily number of trauma admissions we fitted several Poisson time series regression models. Poisson regression is part of the Generalized Linear Model (GLM) framework, alongside traditional linear regression and logistic regression, when the data under study are counts. Poisson regression has the natural logarithm as the link function, and for clinical interpretation regression results must thus be back transformed, resulting in a multiplicative model.

To model the possibly non-linear long-term temporal trend in the daily counts we applied the Generalized Additive Models (GAM) framework [23]. GAM is a natural extension of GLM to allow for non-linear associations. Rather than fit linear terms of time *t* we fit a smooth function *s(t)*, for example using splines. The optimal spline can be found using the Generalized Cross Validation criterion (GCV). Potential cyclic components in the data were modelled using Fourier series [24]. The Fourier series expansion theorem states that any repeating signal with time period *T* can be fitted using a linear combination of sufficiently many sine and cosine functions.

The resulting statistical model has a count variable as outcome and allows for the simultaneous estimation of both a possible non-linear long-term trend and several layers of cyclic patterns, such as yearly and weekly effects, as well as standard covariates. The various models for seasonality in trauma admissions are described below.

#### Poisson time series regression with Fourier series

Modelling both the yearly and weekly cyclic patterns in the data using Fourier series results in the model

$$\begin{array}{l} \ln[E(n_{t})]=a_{0}+s(t)+\sum_{k=1}^{K_{y}}[a_{k}\cos(\frac{2\pi kt}{T_{y}})+b_{k}\sin(\frac{2\pi kt}{T_{y}})]\\ +\sum_{k=1}^{K_{w}}[\alpha_{k}\cos(\frac{2\pi kt}{T_{w}})+\beta_{k}\sin(\frac{2\pi kt}{T_{w}})], \end{array}$$ (1)

with *n*_*t*_ the number of trauma admissions at time *t*, *T*_*y*_ = 365 and *T*_*w*_ = 7.

#### Daylight and weather based model

In order to explore whether seasonality can be explained by meteorological variables alone, we replaced the Fourier series terms, that is, the trigonometric functions, in model (1) representing the yearly cyclic effect with various daylight and weather variables, resulting in the model

$$\ln[E(n_{t})]+a_{0}+s(t)+\sum_{i}f_{i}(w_{t}^{i})+\sum_{k=1}^{K_{w}}[\alpha_{k}\cos(\frac{2\pi kt}{T_{w}})+\beta_{k}\sin(\frac{2\pi kt}{T_{w}})],$$ (2)

with $w_{t}^{i}$, *i* = 1…5, the five variables described previously, at time *t*. In order to establish the association between each of the predictors and the outcome, B-splines were fitted to estimate the functions $f_{i}(w_{t}^{i})$. These were then replaced with linear or piecewise linear functions where applicable, based on visual inspection of the GAM plot and Akaike’s Information Criterion (AIC) model comparison statistics [25]. In piecewise linear regression the independent variables are partitioned into subintervals of the observed range, with boundaries between intervals separated by breakpoints, and individual straight lines fitted on each subinterval.

#### Combined temporal and daylight and weather based model

Acknowledging that neither Fourier series nor meteorological variables might be sufficient to capture all detail in the seasonality component of trauma admissions, we included both in a combined model. This model allowed for a long-term non-linear increase, five daylight and weather covariates, a component of yearly cyclic effects unexplained by the suggested weather covariates, and a week pattern, resulting in the following model;

$$\begin{array}{l} \ln[E(n_{t})]=a_{0}+s(t)+\sum_{i}f_{i\;}(w_{t}^{i})+\\ \sum_{k=1}^{K_{y}}[a_{k}\cos(\frac{2\pi kt}{T_{y}})+b_{k}\sin(\frac{2\pi kt}{T_{y}})]+\sum_{k=1}^{K_{w}}[\alpha_{k}\cos(\frac{2\pi kt}{T_{w}})+\beta_{k}\sin\frac{2\pi kt}{T_{w}})] \end{array}$$ (3)

B-splines were used for estimating the functions $f_{i}(w_{t}^{i})$, but were replaced with linear or piecewise linear functions where applicable.

#### Model comparison

To compare the different statistical models, both for choosing the number of trigonometric terms to include in the Fourier series in individual models (1)-(3), and for ordering of the various models, we used AIC [25]. AIC can be viewed as a weighting between parsimony and model fit to the data and is an objective measure of the “goodness” of a model; the lower the AIC, the better the model. Note that it is not the absolute value of AIC which is important, but the relative values between models, and in particular the AIC differences Δ_i_ = AIC_i_-AIC_min_ [26]. The model estimated to be best has Δ_i_≡ Δ_min_≡0. Models with Δ_i_>10 relative to the best model have essentially no support in the data, while models with 0≤Δ_i_≤2 have substantial support [26].

## Results

**Fig 1:**
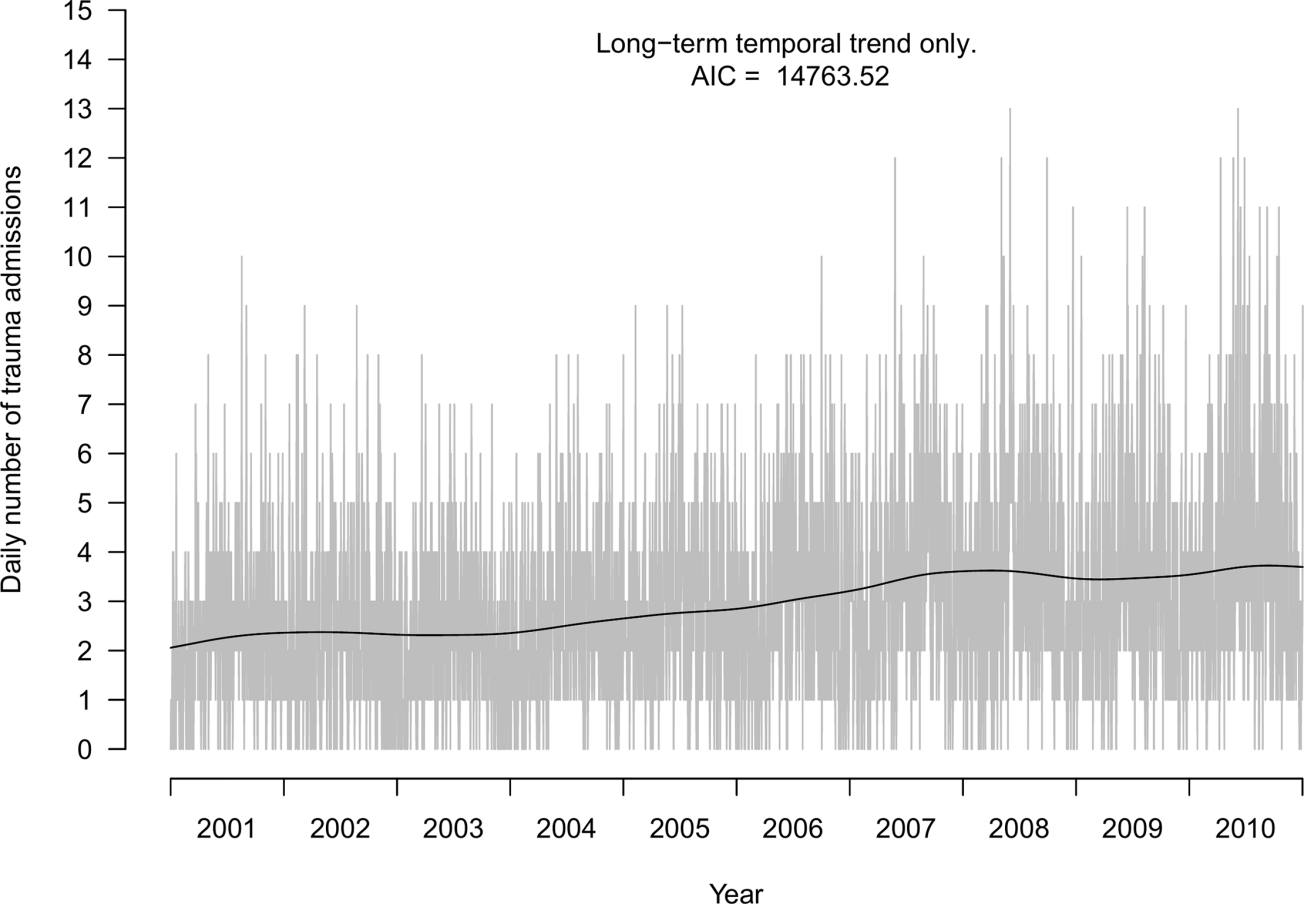
Daily number of trauma admissions. Daily number of trauma admissions at OUH Ullevål 2001–2010. Long-term trend from Generalized Additive Model superimposed. A day is defined as the 24-h time interval starting at 06:00 local time on a given date.

During the 10-year observation period there were 10,726 trauma admissions. There were strong indications of a long-term non-linear increase in the daily number of trauma admissions (Fig 1). Fitting a GAM for the long-term temporal trend while ignoring the yearly variations and the weekly cyclic pattern resulted in the model superimposed in Fig 1 (AIC 14,763.5).

### Temporally explained seasonality

**Fig 2:**
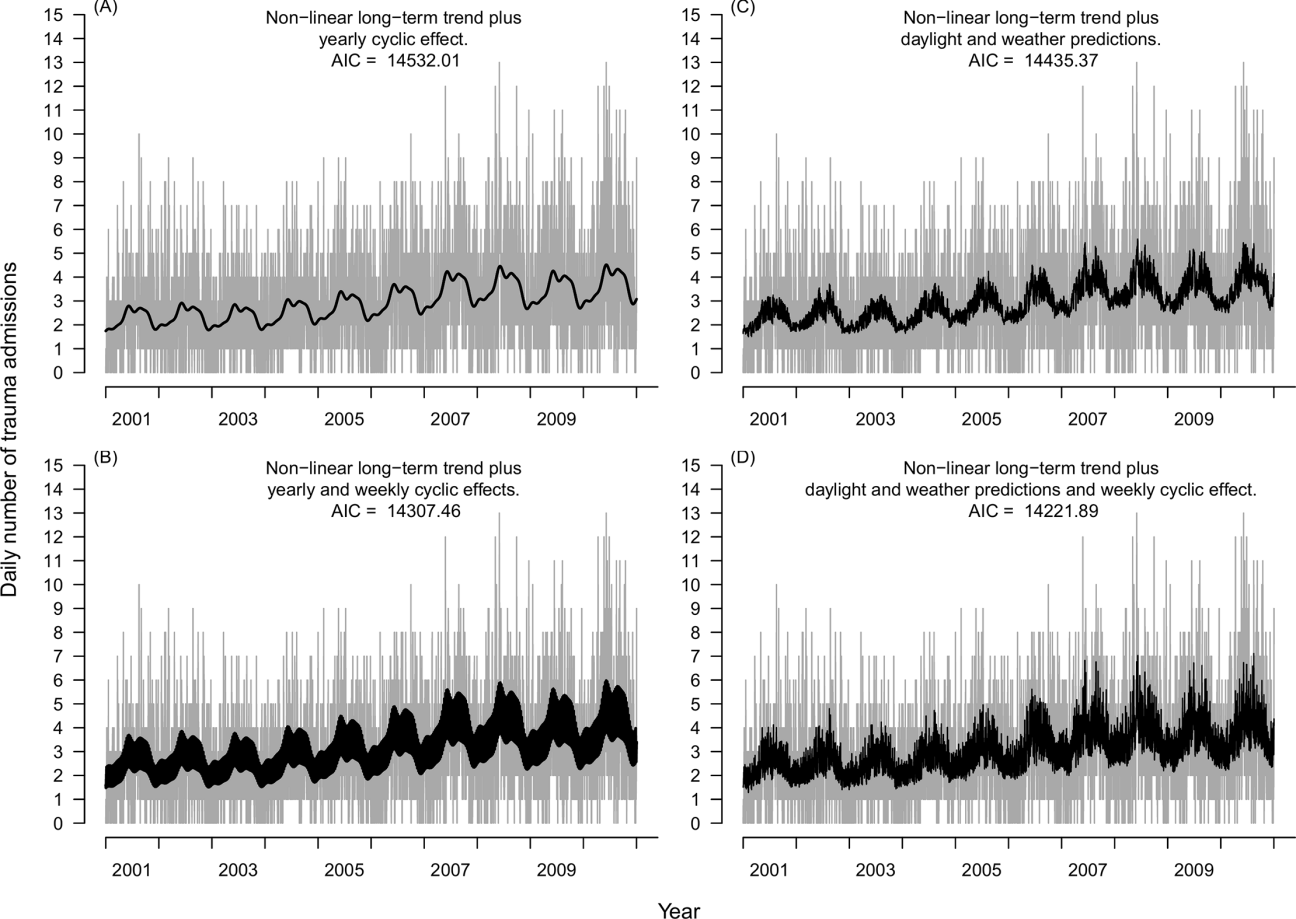
Model fits to daily number of trauma admissions. Daily number of trauma admissions at OUH Ullevål 2001–2010 (grey) with fit from various statistical models superimposed (black). Seasonal models using Fourier series (left column) or daily daylight and weather variables (right column), without an additional week effect (top row) or with an additional week effect added (bottom row).

**Fig 3:**
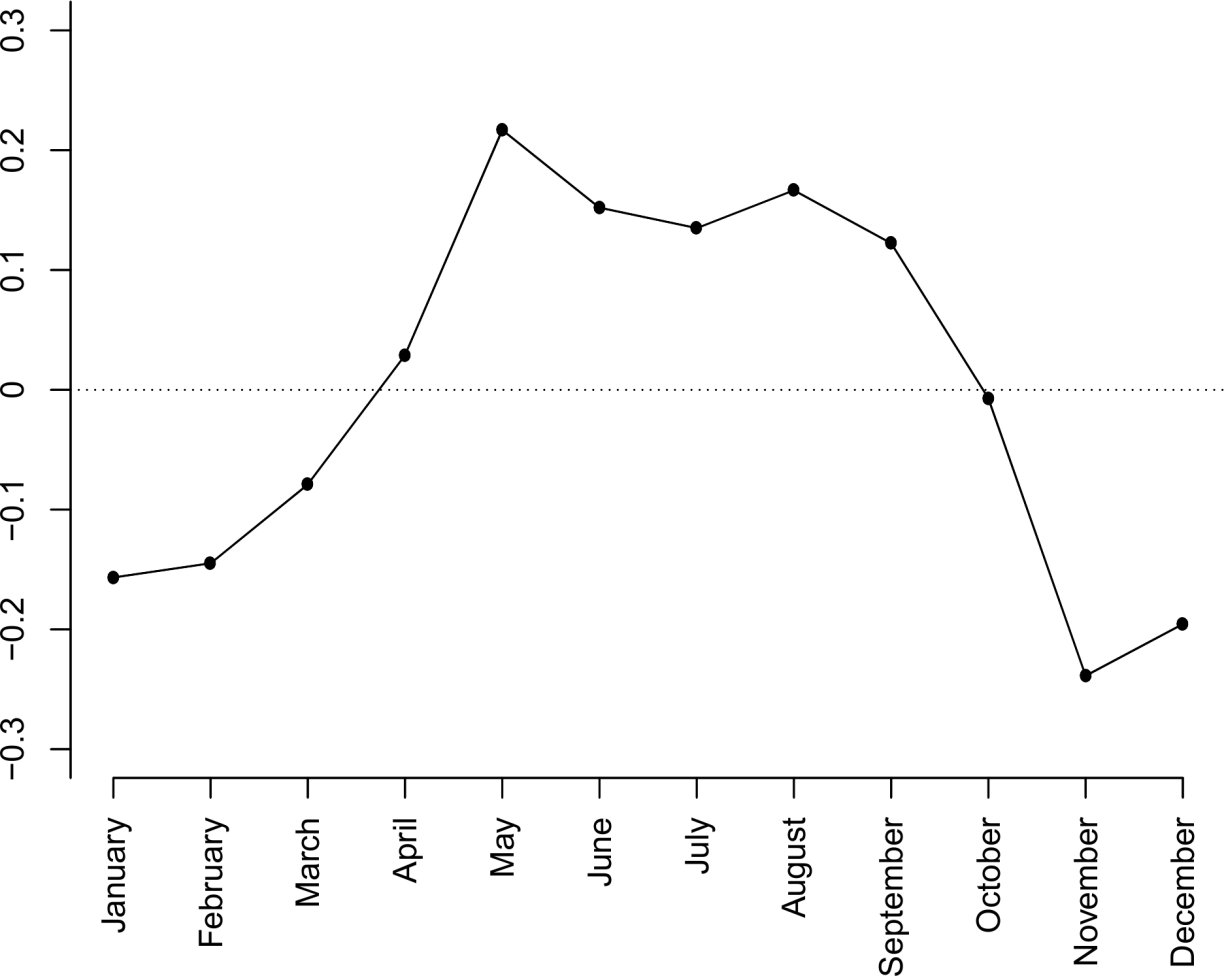
Seasonal component. Estimated seasonal component by trigonometric functions for daily trauma admission across the 10 years 2001–2010.

We fitted both yearly and weekly cyclic effects by Fourier series (model 1), using *K*_*y*_ = 12 and *K*_*w*_ = 7 trigonometric functions for their respective patterns. The model with a long-term increase only (Fig 1) was outperformed by a model with an additional yearly cyclic pattern (Fig 2A; AIC 14,532.0), which was further significantly improved by adding a weekly cyclic pattern (Fig 2B; AIC 14,307.5). Thus, there was strong evidence of both a yearly cyclic effect and a weekly cyclic effect in the number of trauma admissions. Estimated changes in admission numbers throughout the year showed a high in May and a low in November (Fig 3).

### Daylight and weather explained seasonality

**Fig 4:**
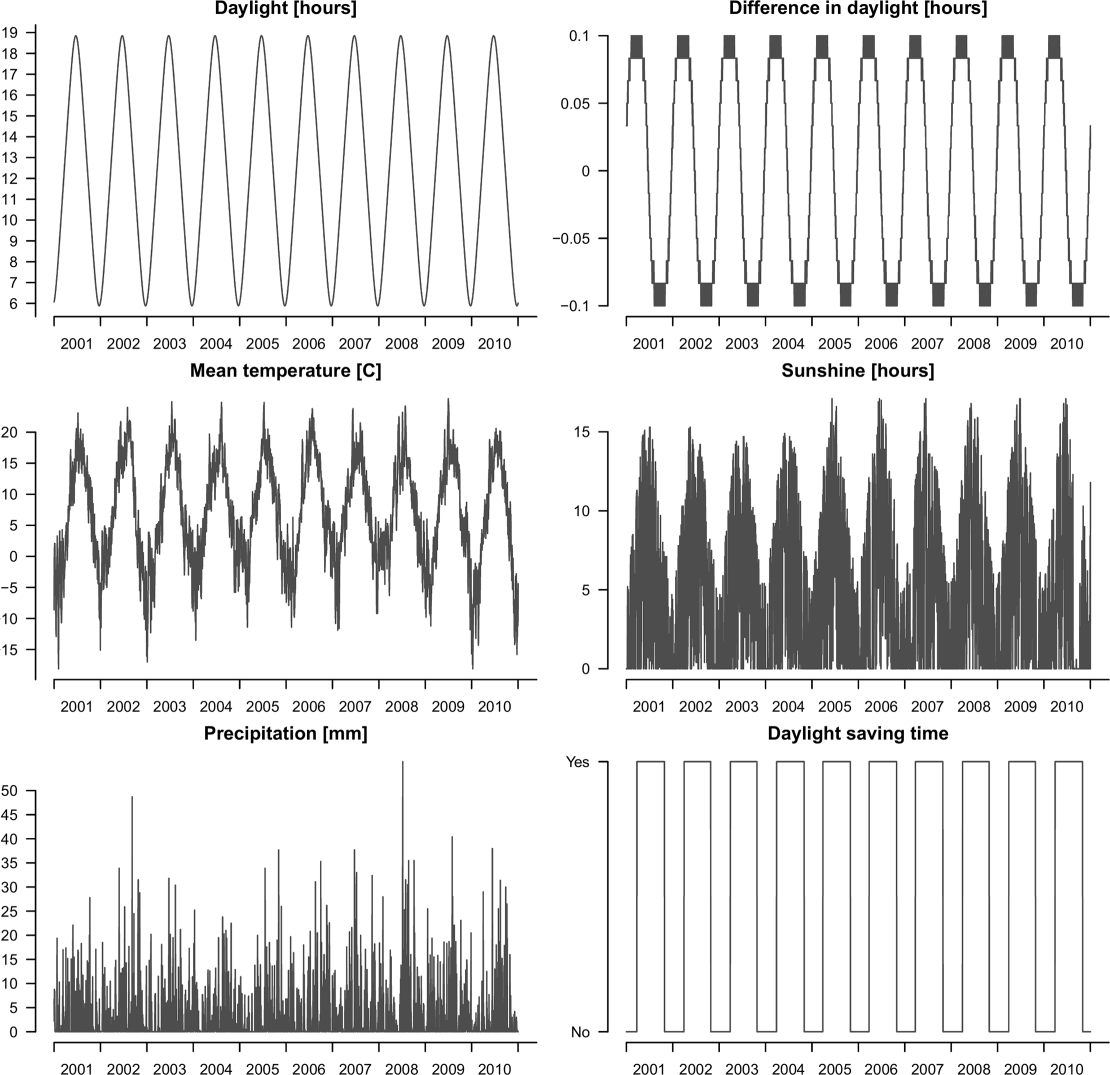
Predictor values. Observed values of daylight and weather variables during the 10 year period 2001–2010.

The values of the five predictors varied strongly throughout the year (Fig 4).

The Spearman correlation between the five predictors ranged from <0.01 (between daylight hours and difference in daylight hours and mm precipitation) to 0.81 (between daylight hours and daily mean temperature), with a median of the absolute values of 0.11 (Table 1).

**Table 1:** Correlation between predictors.

	Daylight [hours]	Difference in daylight [hours]	Sun [hours]	Mean temperature [°C]	Precipitation [mm]
Daylight [hours]	1	<0.01	0.51	0.81	0.04
Difference in daylight [hours]		1	0.02	-0.39	-0.13
Sun [hours]			1	0.46	-0.19
Mean temperature [°C]				1	0.11
Precipitation [mm]					1

We replaced the Fourier modelling of the observed yearly cyclic effect with daylight and weather variables. Fitting a GAM allowing for non-linearity in the association between each of the five variables and the outcome demonstrated a strong indication of non-linearity in the association between day-to-day daylight difference and number of daily trauma admissions (S1 Fig). The association could be well approximated by a piecewise linear model with two breakpoints. Searching through all possible piecewise linear models for breakpoints in [-0.1, 0] and [0, 0.1] hours, respectively, resulted in a well-defined global minimum for the breakpoints -0.067 hours and 0.031 hours. Fitting this piecewise linear model, and still allowing for a smooth fit of time as shown in Fig 1, resulted in a daylight and weather explained model that significantly outperformed its Fourier counterpart (Fig 2C; AIC 14,435.4). Adding a weekly component modelled by trigonometric functions further improved on this (Fig 2D; AIC 14,221.9). That is, a daylight and weather model performed significantly better than a purely temporal model of seasonality.

### Combined seasonality model

**Fig 5:**
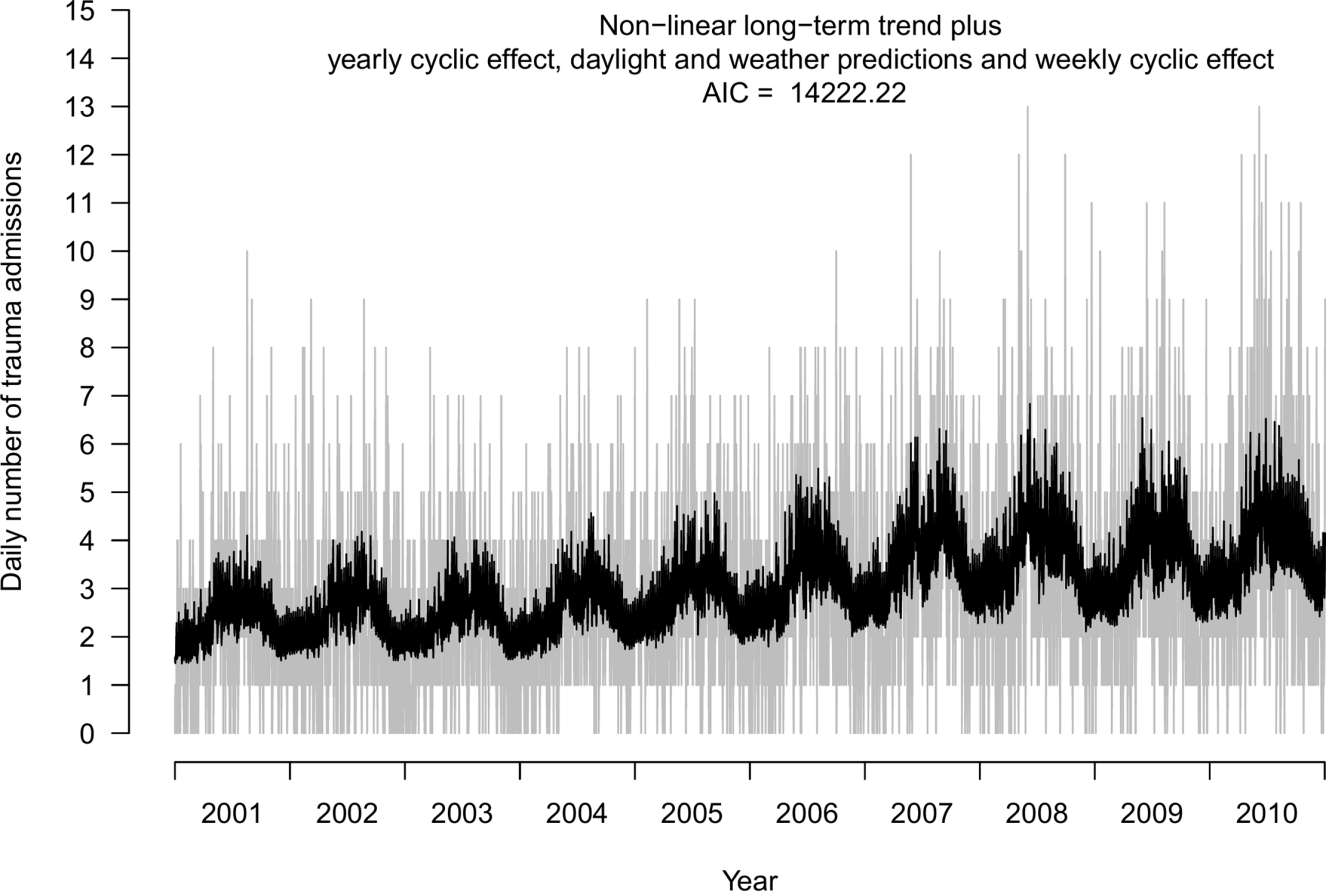
Combined model fitted to daily number of trauma admissions. Daily number of trauma admissions at OUH Ullevål 2001–2010. Combined Fourier and daylight and weather model with cyclic weekly pattern.

The Fourier based seasonality models (Fig 2A and 2B) and the daylight and weather covariate-based models (Fig 2C and 2D) have somewhat different visual appearances. While the meteorological model performed better than the purely temporal model, some of the seasonal variation might not be fully explained by the five included covariates alone. To explore this, we fitted a combined model with daylight and weather variables as well as a Fourier series for potential unexplained surplus seasonality. A cyclic week component was also included. This combined model (Fig 5; AIC 14,222.2) had an AIC comparable to that of the purely meteorological model with the week component (Fig 2D), but also included a low amplitude sine function with period $\frac{2\pi t}{T_{y}}$, that is, one single period.

### Weekly pattern

**Fig 6:**
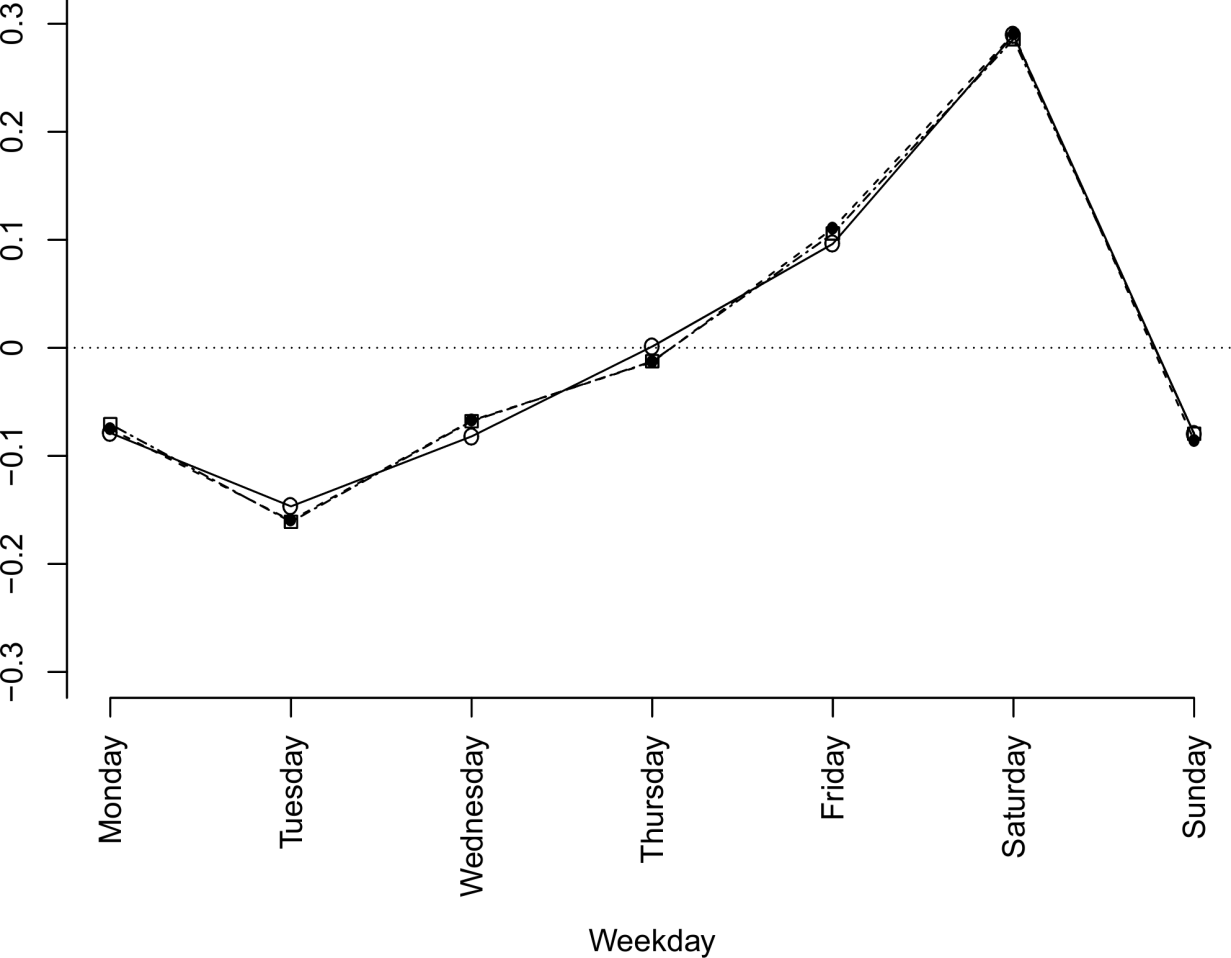
Weekly pattern. Estimated weekly pattern in daily trauma admission across all 572 weeks in the 10 year sample 2001–2010 for the three models displayed in Figs 2B, 2D and 5.

The weekly cyclic component estimated across all weeks in the 10-year observation period for each of the three different modelling approaches is shown in Fig 6. Estimated weekly variations were very similar for the various seasonality models, indicating that the week effect is an independent temporal pattern in the data, unaffected by choice of modelling approach for the seasonal effect.

### Importance of daylight and weather variables

Coefficient estimates for the optimal model based on the measurement data are shown in Table 2. Crude 95% CIs are calculated as ±1.96∙SE. As Poisson regression has the logarithm as the link function we also present the backtransformed results of the corresponding multiplicative model with incidence rate ratios. Each exponentiated coefficient is the multiplicative term to use for calculating the estimated number when a given covariate increases by 1 unit. In the case of categorical variables, the exponentiated coefficient is the multiplicative term relative to the base level for that variable. The exp(Intercept) is the baseline rate, and all other estimates are relative to it. The intercept corresponds to a day with zero degrees, zero sunlight and zero precipitation.

**Table 2:** Poisson regression results.

	Measurement data			Standardized data		
	Logarithmic scale / additive model		Back transformed / multiplicative model*	Logarithmic scale / additive model		Back transformed / multiplicative model*
	Coefficients Estimate (95% CI)	p-value	Incidence rate ratios Estimate (95% CI)	CoefficientsEstimate (95% CI)	p-value	Incidence rate ratios Estimate (95% CI)
(Intercept)	0.376 (0.136, 0.615)	0.002	1.456 (1.145, 1.850)	1.066 (1.038, 1.094)	<0.001	2.904 (2.823, 2.987)
Daylight duration [hours]	0.014 (0.003, 0.025)	0.013	1.014 (1.003, 1.026)	0.061 (0.013, 0.110)	0.013	1.063 (1.013, 1.116)
Difference in daylight duration [hours] ††						
*Effect below lower breakpoint*	-2.734 (-6.209, 0.739)	0.123	0.065 (0.002, 2.094)	-0.207 (-0.470, 0.056)	0.123	0.813 (0.625, 1.058)
*Added effect b/w breakpoints*	5.907 (2.487, 9.327)	<0.001	367.6 (12.0, 1124)	0.404 (0.170, 0.639)	<0.001	1.499 (1.186, 1.894)
*Added effect above upper breakpoint*	-2.164 (-4.181, -0.147)	0.035	0.115 (0.015, 0.863)	-0.057 (-0.110, -0.004)	0.035	0.945 (0.896, 0.996)
Sunshine [hours]	0.015 (0.010, 0.021)	<0.001	1.016 (1.010, 1.021)	0.072 (0.047, 0.098)	<0.001	1.075 (1.048, 1.103)
Mean temperature [°C]	0.008 (0.002, 0.014)	0.005	1.008 (1.002, 1.014)	0.067 (0.020, 0.114)	0.005	1.070 (1.020, 1.121)
Precipitation† [mm]						
*(0–5]*	-0.069 (-0.117, 0.021)	0.005	0.933 (0.889, 0.980)	-0.069 (-0.117, 0.021)	0.005	0.933 (0.889, 0.979)
*(5–10]*	-0.093 (-0.171, 0.015)	0.020	0.911 (0.843, 0.985)	-0.093 (-0.171, 0.015)	0.020	0.911 (0.843, 0.985)
*(10*,*→)*	-0.056 (-0.140, -0.028)	0.193	0.946 (0.869, 1.029)	-0.056 (-0.140, 0.028)	0.193	0.946 (0.869, 1.029)

Results from a multiple Poisson time series regression model with number of daily trauma admissions as outcome and various daylight and weather variables as predictors.

*As Poisson regression has the logarithm as the link function these are backtransformed results (exponentiated coefficients) of a multiplicative model with incidence rate ratios.

†Reference category 0, i.e. no precipitation.

†† Breakpoints at -0.067 and 0.03.

While the daylight and weather variable model outperformed the Fourier models, the included predictors are measured on very different scales. Refitting the final model using standardized continuous predictors allows for the comparison of the relative importance of the various predictors (Table 2). Using standardized data the intercept represents a day with mean value for all covariates included in the model. Hours of sunshine had a stronger effect on the increase in number of admissions than the mean temperature. The strongest effect however was that of difference in daylight hours. The effect of difference in daylight on daily trauma admissions was non-significant below the lower breakpoint (-0.067 hours ≈ 4 minutes less daylight than the day before), but was associated with a strong increase in the number of trauma admissions between this lower breakpoint and the upper breakpoint (0.031 hours ≈ 1.9 minutes more daylight than the day before). Above this upper breakpoint the effect of difference in daylight tailed off.

## Discussion

The steady but non-linear increase in trauma admissions at Oslo University Hospital (OUH) during the 10-year observation period has been described previously [18], and the observed significant seasonal cyclic pattern of trauma admissions is in agreement with known variations [4]. Seasonality has often been explored using aggregated monthly data. However, with an underlying steady increase in number of trauma admissions estimating seasonal effects by pooling months from different years together might overestimate the natural variation for individual months, and also underestimate the precision of the seasonality component. Our methodological approach allows for more detail and continuous adjustment both between and within months.

Studies exploring seasonal effects tend to treat months and years as separate units in time [3, 7, 8, 27]. The observed long-term non-linear increase in daily number of trauma admissions in this study underpins that using months as the unit of analysis cannot automatically be recommended, as this will effectively imply turning continuous time into a categorical variable, potentially masking clinically valuable information. Categorising continuous predictors in multiple regression models has been thoroughly examined and repeatedly argued against in the statistical literature [28–31]. The modelling approach applied here avoided this while still adjusting for the long-term non-linear changes.

While seasonal decomposition of time series models is well known in the literature, traditional time series modelling tends to focus on forecasting capabilities rather than model building, covariate exploration and hypothesis testing. The latter aspects are often the focus in health research. An alternative to traditional time series analysis of temporal data is Poisson time series regression. A comparison of time series analysis and Poisson regression for detecting a shift in change in rates of child injuries after an intervention in New York deemed Poisson regression an attractive alternative to time series analysis [32]. Time series Poisson regression analysis has been used to analyse the association between dengue fever and weather in China [33], and its use in environmental epidemiology has been explored [34].

Analysing seasonality through Poisson regression by including a few select sine and cosine functions has been suggested previously [24], and this approach has been applied for analysing seasonality in road traffic injuries [35]. The idea has recently been extended to fit optimal Fourier series to model seasonality in suicide [17]. This Additive Fourier Poisson time series regression model adjusts for both non-cyclic and cyclic temporal phenomena, e.g. a non-linear long-term increase and seasonality, without the need for data aggregation or categorisation of time into culturally common units.

Fitting several statistical models our study demonstrated that multiple daylight and weather covariates taken together predicted seasonality in trauma admissions better than a purely temporal model of yearly variations, when adjusting for both a long-term non-linear increase in admissions and the well-known short-term weekly cyclic effect.

The strongest of the weather predictors was the change in daylight hours from the previous day, i.e. the daylight gradient. In the field of reproduction hormone variations throughout the year has been studied thoroughly. Data from studies on Siberian hamsters indicate that the photoperiodic time measurement system responds not only to the length of the day, but also to the direction of change in day length [36, 37]. Naturally increasing day lengths is more reproductively stimulatory than abrupt transfer to static long day lengths [38]. Intermediate photoperiods are reproductively inhibitory if preceded by longer photoperiods, but do not inhibit reproductive physiology if preceded by equivalent or shorter day lengths [11]. Human functional MRI data has demonstrated that the neuronal response to risk-taking behaviour could be enhanced by bright-light therapy; however the stimulus used was constant throughout the intervention [6]. We have not found human studies using cyclic light stimuli.

Our study demonstrates that human behaviour is also affected by daylight and weather variables, and that this can explain observed seasonal effects in trauma admissions. Future research should explore not only same-day factors, but also lagged associations. Notably, Norway is located far north in Europe, with large seasonal effects in the weather. Temperatures typically vary from -20°C in winter to 20°C in summer, accompanied by heavy rainfall in spring and autumn. Variations in daylight hours are also large. In Northern Norway the range goes all the way from total darkness during winter to never-ending daylight in summer. In the capital Oslo the range is from 5:53 hours of daylight in winter through 18:51 hours during summer [19]. With such strong seasonal variations countries like Norway might be particularly well suited for studies of seasonal effects in humans.

## Conclusion

Seasonality in trauma admissions is a well-known phenomenon, probably caused by a multitude of sociocultural and neuroendocrine factors. Our analyses indicate that this effect can be directly ascribed to various daily measures of daylight and weather. Long- and short-term weather forecasts could be a valuable resource of information for health care planners. Further, while day-to-day daylight and weather changes might be a resource demanding addition to planning, length of day, and whether it increases or decreases, is very predictable, and easy to use for long-term planning.

## Supporting information

S1 Fig Spline fits. Estimated splines from full GAM model for all four continuous meteorological variables. Dashed lines are 95% confidence intervals.
(TIF)
