# Supplementary figures and images for: Seasonality in trauma admissions – Are daylight and weather variables better predictors than general cyclic effects?
Source: PLoS One. 2018 Feb 9;13(2):e0192568. doi: 10.1371/journal.pone.0192568 (PMC5806884; doi:10.1371/journal.pone.0192568)

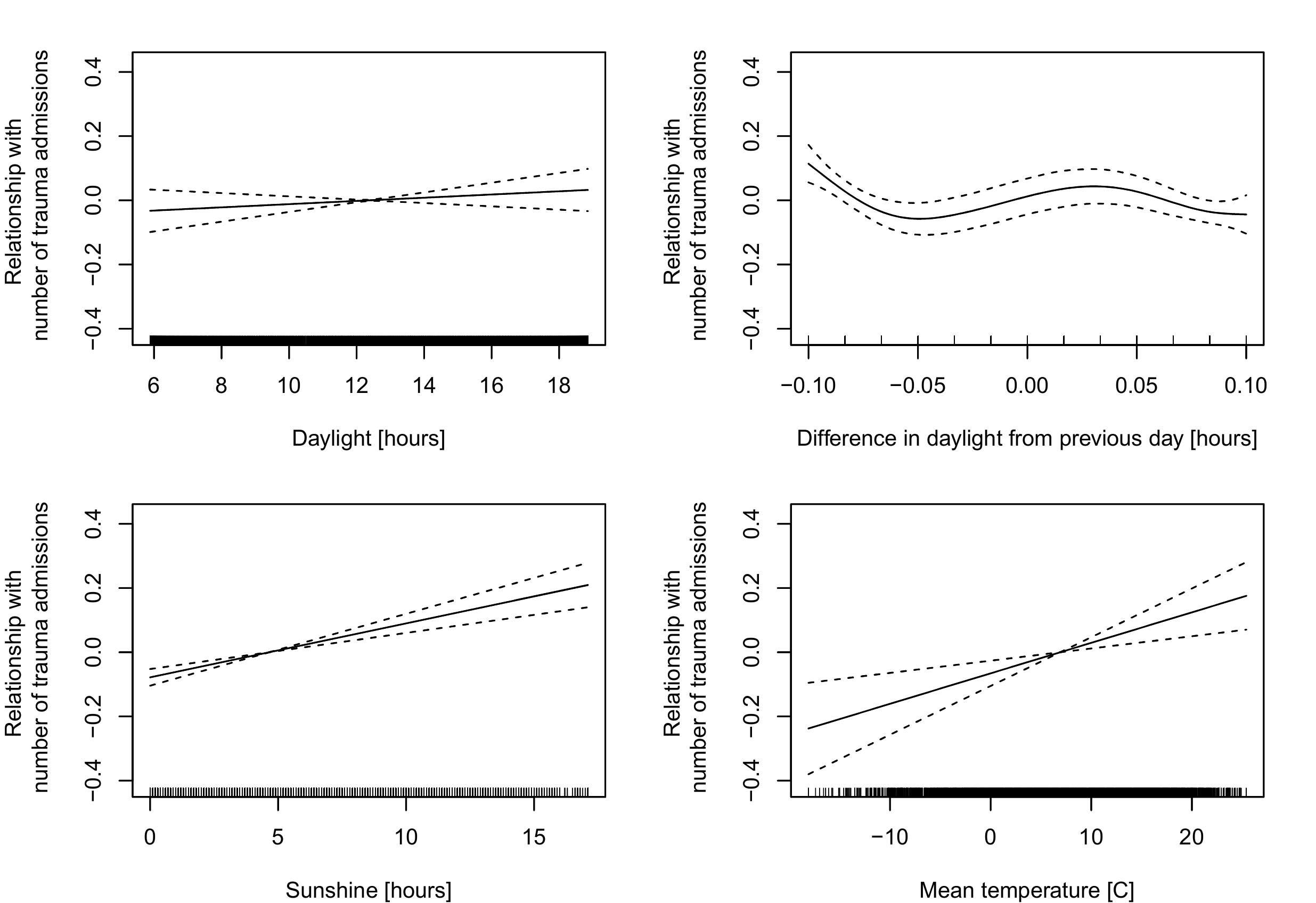

Supplement: S1 Fig — Estimated splines from full GAM model for all four continuous meteorological variables. Dashed lines are 95% confidence intervals. (TIF) [file pone.0192568.s001.tif]
